# Diet-induced hyperhomocysteinemia causes sex-dependent deficiencies in offspring musculature and brain function

**DOI:** 10.3389/fcell.2024.1322844

**Published:** 2024-03-15

**Authors:** Joanna Suszyńska-Zajczyk, Łukasz Witucki, Joanna Perła-Kaján, Hieronim Jakubowski

**Affiliations:** ^1^ Department of Biochemistry and Biotechnology, Poznań University of Life Sciences, Poznań, Poland; ^2^ Department of Microbiology, Biochemistry & Molecular Genetics, Rutgers University, New Jersey Medical School, International Center for Public Health, Newark, NJ, United States

**Keywords:** hyperhomocysteinemia, pregnancy outcomes, offspring, high-Met diet, behavior and cognition

## Abstract

Hyperhomocysteinemia (HHcy), characterized by elevated homocysteine (Hcy) levels, is a known risk factor for cardiovascular, renal, and neurological diseases, as well as pregnancy complications. Our study aimed to investigate whether HHcy induced by a high-methionine (high-Met) diet exacerbates cognitive and behavioral deficits in offspring and leads to other breeding problems. Dietary HHcy was induced four weeks before mating and continued throughout gestation and post-delivery. A battery of behavioral tests was conducted on offspring between postnatal days (PNDs) 5 and 30 to assess motor function/activity and cognition. The results were correlated with brain morphometric measurements and quantitative analysis of mammalian target of rapamycin (mTOR)/autophagy markers. The high-Met diet significantly increased parental and offspring urinary tHcy levels and influenced offspring behavior in a sex-dependent manner. Female offspring exhibited impaired cognition, potentially related to morphometric changes observed exclusively in HHcy females. Male HHcy pups demonstrated muscle weakness, evidenced by slower surface righting, reduced hind limb suspension (HLS) hanging time, weaker grip strength, and decreased activity in the beaker test. Western blot analyses indicated the downregulation of autophagy and the upregulation of mTOR activity in HHcy cortexes. HHcy also led to breeding impairments, including reduced breeding rate, in-utero fetal death, lower pups’ body weight, and increased mortality, likely attributed to placental dysfunction associated with HHcy. In conclusion, a high-Met diet impairs memory and cognition in female juveniles and weakens muscle strength in male pups. These effects may stem from abnormal placental function affecting early neurogenesis, the dysregulation of autophagy-related pathways in the cortex, or epigenetic mechanisms of gene regulation triggered by HHcy during embryonic development.

## 1 Introduction

Homocysteine (Hcy), a sulfur-containing amino acid, integral to methionine (Met) metabolism (Obeid and Herrmann, 2006), is derived solely from dietary Met in mammals (Jakubowski, 2019). Hcy elevated above 15 μM, termed hyperhomocysteinemia (HHcy), is an independent risk factor of cardiovascular (Huang et al., 2012) and neurodegenerative diseases (Seshadri et al., 2002; Cordaro et al., 2021) and may adversely affect various stages of reproduction (Akamine et al., 2021), such as decreased semen parameters (morphology, sperm count, and/or motility), fetal congenital malformation, increased abortion rate, gestational hypertension, gestational diabetes, or low birth weight (de la Calle et al., 2003; Makedos et al., 2007). Moreover, recent studies implicated HHcy as a risk factor for neurological and psychiatric disorders (Wyse et al., 2020).

The causes of Hcy accumulation include genetic aberrations in the remethylation or transsulfuration pathways in Met metabolism, or nutritional deficiencies such as inadequate supplementation of vitamin B12, B6, and folate, or prolonged methionine intake (Smith and Refsum, 2016). In humans, oral administration of free Met leads to a dose-dependent increase in plasma total homocysteine (tHcy) concentrations within a few hours (Verhoef and de Groot, 2005). tHcy increases up to 10-fold in mice on a high-Met diet compared to those on a control diet (Jakubowski et al., 2009). Adding 0.5%–1% Met to drinking water is a recognized method for inducing moderate HHcy in murine models, used by our research group (Borowczyk et al., 2012; Suszyńska-Zajczyk et al., 2014a; Suszyńska-Zajczyk et al., 2014b; Suszyńska-Zajczyk et al., 2014c; Suszyńska-Zajczyk et al., 2014d) and other research groups (Dayal et al., 2008; Dayal and Lentz, 2008; Xu et al., 2011; Kovalska et al., 2019).

HHcy negatively impacts human sperm quality and oocyte maturation (Peñagaricano et al., 2013; Jia et al., 2019) and alters epigenetic regulation (Jia et al., 2016; McKee et al., 2018; Clare et al., 2019; Perła-Kaján and Jakubowski, 2019; Van Winkle and Ryznar, 2019). In addition to the effects on fertility and pregnancy outcomes, Hcy accumulation is a strong determinant for congenital neural tube defects (Afman et al., 2005), other central nervous system (CNS) alterations (Mills et al., 1996), and additional congenital malformations (i.e., heart defects and oral clefts) (Iacobazzi et al., 2014). Additionally, HHcy dysregulates homeostasis, which can have lifelong consequences (Chmurzynska, 2010; Boersma et al., 2014; Yajnik et al., 2014). Generally, changes in the uterine environment during pregnancy have the potential to induce long-lasting alterations in both behavior and physiology. Maternal Hcy concentration also plays a causative role in determining fetal growth and birth size (Yajnik et al., 2014) and is an important factor causing prenatal stress and the impairment of nervous system development in fetuses, newborns, and early ontogenesis, as well as complications in adulthood (Arutjunyan et al., 2021). Due to placental blood flow disturbance, maternal HHcy can contribute to serious impairments in the embryonic brain development and its maturation in early postnatal ontogenesis, as demonstrated in rodents (Shcherbitskaia et al., 2021; Vasilev et al., 2023), leading to a decrease in memory and locomotive activity (Blaise et al., 2007; Shcherbitskaia et al., 2020). In humans, elevated preconception tHcy is inversely associated with lower psychomotor and cognitive development scores in infants and children (Murphy et al., 2017) and a higher risk of psychological problems in childhood (Ars et al., 2019). It was also found that HHcy during pregnancy is associated with an increased risk of schizophrenia in adulthood due to placental impairment (Brown et al., 2007).

Oxidative stress and vascular dysfunction both during pregnancy and postnatal development are well-established mechanisms of HHcy pathology by which behavior and brain homeostasis in offspring may be altered. Another mechanism may involve the dysregulation of the mammalian target of rapamycin (mTOR) signaling in HHcy, which warrants further investigation. Several studies have shown that increased Hcy level upregulates mTOR (Tripathi et al., 2016; Khayati et al., 2017). mTOR is a conserved serine/threonine kinase that is involved in a variety of processes including regulation of growth, survival, motility, cell division, transcription, and protein synthesis. Dysregulation of mTOR constitutes the primary cause of the development of several neurological impairments (Poddar et al., 2023), including Huntington’s disease (HD) (Roscic et al., 2011), AD (Grünblatt et al., 2023), autism (Winden et al., 2018), and other neurological and psychiatric disorders (Bockaert and Marin, 2015). Increased activation of mTOR results in increased dendrite branching, higher numbers of immature filopodia-like protrusions in dendrites, and a decrease in the density of mature dendritic spines, leading to cognitive impairments (Troca-Marín et al., 2014). mTOR mediates several different aspects of cerebrovascular dysfunction, including blood–brain barrier (BBB) breakdown, cerebral hypoperfusion, reduced cerebrovascular reactivity, impaired neurovascular coupling (Jahrling et al., 2018), and primary vascular impairment (Van Skike et al., 2021). Autophagy is regulated by mTOR, and aberrations in signaling through both pathways have been identified as key drivers in the development of various human diseases, such as cancer and neurodegenerative disorders (Perluigi et al., 2015). In murine models, it was proven that the absence of autophagy results in neurotoxicity (Hara et al., 2006; Komatsu et al., 2006). Recent studies have shown that HHcy inhibits autophagy via the activation of (mTOR), which worsens cognitive performance in AD mouse models (Witucki and Jakubowski, 2023a; Witucki and Jakubowski, 2023b; Witucki et al., 2023).

Widely recognized, parental nutrition influences both reproductive performance and fetal growth and development. Moreover, the reproductive period stands as a crucial window for shaping the potential risks of chronic diseases in the offspring’s later life. Our primary goal was to evaluate the hypothesis that parental HHcy, induced by a high-Met diet, worsens cognition and behavioral performance in offspring during the neonatal up to the adolescent stage. Additionally, we explored alterations in the mTOR and autophagy pathways in these individuals. Despite recognizing HHcy as a prevalent condition in pregnancy pathology and behavioral disorders, many underlying mechanisms remain unknown. In this study, we investigated the effects of chronic parental HHcy on offspring to correlate brain structure changes and mTOR/autophagy dysregulation with the observed locomotor and cognitive impairments.

## 2 Materials and methods

### 2.1 Mice, diet, and breeding

**FIGURE 1:**
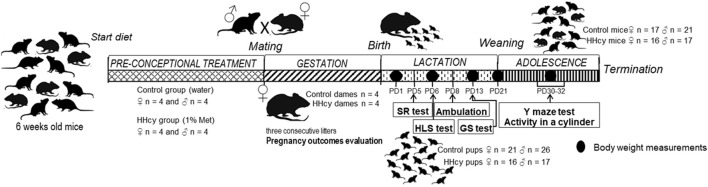
Experimental workflow.

Eight females and eight males of C57BL/6J mice were divided into two experimental groups: control and HHcy. Mice in both groups were fed a normal rodent chow. In the experimental group, HHcy was induced by providing 6-week-old mice with 1% methionine in drinking water (high-Met diet) and continued during the whole experiment, while the control group received pure water. Both groups had *ad libitum* water access with similar consumption. Then, 10-week-old mice were mated and caged in pairs (four pairs for each group). We allowed three constitutive breeds for each pair. The pregnancy duration was monitored, females were weighed twice a week, and the interpregnancy interval was measured. The total number of born offspring, number of stillborn fetuses, and the survival rate after postnatal day (PND) 3 were observed. The diet was maintained throughout lactation, during which neonates underwent a battery of behavioral assessments. After weaning, the diet persisted, and 1-month-old mice were subjected to the Y-maze test and beaker test (BT). Offspring body weight was measured on PNDs 1, 6, 13, 30, and 40—at the termination using a precision balance for live rodents. The experimental timeline, detailing HHcy induction before pregnancy and progeny behavioral tests, is presented in Figure 1.

All animal procedures were approved by the Local Ethics Committee for Animal Experiments in Poznan, Poland (approval no. 83/2016).

### 2.2 Total Hcy assay

Urinal total Hcy (tHcy) was assayed in parents and offspring using HPLC-based method with post-column derivatization as described previously (Chwatko and Jakubowski, 2005; Jakubowski, 2008).

### 2.3 Testosterone level analysis

Sires’ urinal testosterone levels were measured using an immunoenzymatic reaction–testosterone ELISA Kit (Abcam), according to the manufacturer’s instructions. 25 μL urine and standards were added to a multi-well plate. The testosterone–HRP conjugate (100 µL) was incubated at 37°C for 1 h. After washing, 100 µL TMB substrate was added and incubated for 15 min at 37°C. The reaction was stopped, and the absorbance was measured at 450 nm. The optimal standard curve and regression equation were obtained using the CurveExpert Basic program.

### 2.4 Behavioral tests

Pups underwent evaluation for neonatal weight, survival rates (fetal/neonatal/postnatal deaths), and tHcy levels. Subsequently, assessments of reflex and locomotor functions were conducted during infancy, including the surface righting (SR) test on PND 5, hind limb suspension (HLS) test on PND 6, ambulation on PND 9, and grip strength (GS) on PND 13. In adolescence, the effect of HHcy on short-term memory and activity was assessed by studying spontaneous alternation behavior in the Y-maze test (PND 30) and spontaneous locomotor activity in a cylinder (PND 32). Tests were performed before noon, between 8:30 a.m. and 11:30 a.m., to minimize potential diurnal influences on behavioral variations. Pups were briefly separated from the dam for less than 15 min to mitigate temperature fluctuations, hunger-related issues, and separation anxiety. The pups were allowed to rest between the individual tests to ensure that their utmost performance could be achieved during each testing session. Neonatal body weight was measured on PNDs 1, 6, 13, and 30 using a precision balance designed for live rodents. The test results were analyzed within groups, separately for male and female offspring.

#### 2.4.1 Surface righting reflex

The motor ability of a mouse pup to flip onto its feet from a supine position was tested in SR on PND 5 (Dallman and Ladle, 2013). Five-day-old pups were placed on their backs on a paper sheet and held in position for 5 s. After releasing, the time it takes the pup to return to a prone position was recorded. A maximum time of 60 s was given for each trial if needed. In cases where the pup did not attain successful completion, the testing procedure was aborted, and the pup was returned to a home cage. The test was repeated three times on the same day with an intersection interval of 15 min. The average time/score was taken from the three attempts.

#### 2.4.2 Hind limb suspension

Hind limb strength and neuromuscular function were assessed using the HLS test (El-Khodor et al., 2008). Six-day-old pups were gently positioned face down in a 50-mL conical tube with hind legs hanging over the rim, inducing tension until muscle fatigue. The HLS test evaluates two parameters: suspension time (HLS time)—the duration the pup hangs until falling—and suspension score (HLS score). The HLS score, ranging from 0 to 4, assesses hind limb and tail positions. A score of 4 indicates normal separation of hind limbs with a raised tail; 3 suggests weakness and closer hind limbs; 2 shows frequent hind limb contact; 1 reflects weakness with almost constant clasping and a raised tail; and 0 indicates continuous clasping with a lowered tail or an inability to hold onto the tube (Supplementary Table S1; Supplementary Figure S1). Each pup was tested three times, and the average HLS time and score were calculated.

#### 2.4.3 Ambulation

Crawling is a behavior that emerges in neonate mice between PNDs 0 and 5. Following this period, between days 5 and 10, there is a transition as the pups shift from crawling to walking behaviors. To assess the changes in ambulation in HHcy mice, a nine-day-old pup was placed on a flat surface, and ambulation was observed for 3 min (Feather-Schussler and Ferguson, 2016). Pup evaluation used a scale: 0 = no movement, 1 = crawling with asymmetric limb movement, 2 = slow crawling with symmetric limb movement, and 3 = fast crawling/walking.

#### 2.4.4 Grip strength

Grip strength was assessed on PND 13 using a rotating mesh test (Corti et al., 2010). Pups were placed on a 16 × 16 cm wire mesh screen, allowing a 5-s adjustment period. The screen was then slowly rotated horizontally to vertically to test the grasping ability of all four limbs. The falling angle was recorded and used to calculate the average falling angle for each group.

#### 2.4.5 Y-maze

Short-term memory and locomotor activity in 1-month-old mice were assessed using the Y-maze test (Wolf et al., 2016). The Y-maze, with three arms of equal angles, measured 30 cm in length, 20 cm in height, and 5 cm in width each. Mice were placed at the end of one arm and allowed to explore for 9 min. The sequence and number of arm entries were recorded. Spontaneous alternation behavior, indicating spatial working memory, was calculated as a percentage: alternation (%) = [(number of alternations)/(total arm entries–2)] × 100. The total number of arm entries served as a measure of locomotor activity.

#### 2.4.6 Spontaneous locomotor activity in a cylinder (BT)

This test can be used to measure spontaneous movements driven by the mouse’s curiosity to explore (Mann et al., 2015). One-month-old mice were positioned in a 1-L transparent beaker to encourage vertical exploration, and the count of rears made by each mouse was recorded (Fleming et al., 2013). Rears with forelimb wall contacts were scored, defined as a vertical movement with both forelimbs off the floor, with the mouse standing solely on its hind limbs.

### 2.5 Quantification of mTOR and autophagy-related proteins in the cortex

#### 2.5.1 Brain protein extraction

One week after behavioral tests, mice were euthanized by decapitation. Brains were collected, weighted, freshly divided into various regions, and frozen on dry ice. For this analysis, frozen cortexes were pulverized with dry ice using a mortar and pestle and stored at −80°C. Then, proteins were extracted using RIPA buffer (4 v/w, containing protease and phosphatase inhibitors) with sonication (Bandelin SONOPLUS HD 2070) on wet ice (three sets of five 1 s strokes with 1 min cooling interval between strokes). Brain extracts were clarified by centrifugation (15,000 g, 30  min., 4°C), and supernatants having 8–12 mg protein/mL were collected. Protein concentrations were measured with the BCA kit (Thermo Scientific).

#### 2.5.2 Western blot analysis

Proteins were separated by SDS-PAGE on 10% gels (20 mg protein/lane) and transferred to the PVDF membrane (0.2 µm; Bio-Rad, cat.#1620177) for 20 min at 0.1 A, 25 V, using the Trans-Blot Turbo Transfer System (Bio-Rad), as previously described (Witucki et al., 2023). After blocking with 5% bovine serum albumin in 1X tris-buffered saline and 0.1% Tween 20 (TBST, 1 h, RT), the membranes were incubated overnight at 4°C with anti-mTOR (CS #2983), anti-pmTOR Ser2448 (CS, #5536), anti-Atg5 (CS, #12994), anti-Atg7 (CS, #8558), anti-Bcln1 (CS, #3495), anti-p62 (CS, #23214), and anti-Gapdh (CS, #5174). Membranes were washed three times with TBST, 10 min each, and incubated with goat anti-rabbit IgG secondary antibody conjugated with horseradish peroxidase (1 h, RT). Positive signals were detected using the WesternBright Quantum-Advansta K12042-D20 and GeneGnome XRQ NPC chemiluminescence detection system. Band intensity was calculated using the GeneTools program (Syngene). At least *n* = 5 mice per group were assayed in three independent experiments, and mean ± SD values were calculated. The tested proteins are detailed in Supplementary Table S2.

### 2.6 Fixation, tissue processing, and morphometric assessments

For morphometric assessments, the animals were sacrificed under isoflurane anesthesia by trans-cardiac perfusion with 4% paraformaldehyde in 0.1 M phosphate buffer (PBS). Brains were dissected out and immersed in the same fixative for post-fixation for 24 h at 4°C. Tissues were placed in 15% sucrose in PBS, followed by 20% and 30% sucrose in PBS each time overnight or until tissue sank. Brains were embedded in OCT (Fisher Scientific), and coronal sections of the brain were cut into 30 μm thickness using a Leica CM1520 cryostat. A series of slices distant 180 µm from each other were stained according to the standard hematoxylin and eosin protocol. Photomicrographs of at least five sections between bregma −1.80 mm and −2.30 mm for each animal were used for determining the brain parameters: whole brain vertical and horizontal length, hippocampal vertical and horizontal length, the layer thickness of pyramidal neurons of cornu ammonis 1 (CA1), and the granule cells of dentate gyrus ectal limb (DGEC). The area of the brain and hippocampus was calculated using the formula for the area of an ellipse. Representative micrographs of hematoxylin–eosin-stained coronal hippocampal sections from 1-month-old control and HHcy offspring are shown in Supplementary Figure S2.

### 2.7 Statistical analysis

Data were presented as the mean ± SD. Statistical significance for biochemical (tHcy, testosterone), physiological (weight), and morphometric data and most pregnancy outcome measurements was assessed with Student's t-test. Ordinary one-way ANOVA was applied to variables with a normal distribution: Sidak’s multiple comparison test for HLS, GS, Y-maze alteration, or uncorrected Fisher’s test for SR, BT, and intrauterine mortality. The significance level was set at α = 0.05. Graphs and statistical analyses were conducted using GraphPad 8.0 software.

## 3 Results

### 3.1 Pre-conceptional parental urinary total Hcy levels

The high-Met diet significantly elevated urinal total Hcy [µM] by over 5-fold in dams and over 6-fold in sires (Table 1). Furthermore, this diet tended to reduce testosterone levels in HHcy sires compared to control males (1.31 ± 0.47 and 0.85 ± 0.36 ng/mL, respectively).

**TABLE 1:** Mean urinary total tHcy levels (µM) of control and HHcy mice and their offspring.

Sex and age (days)	Control mice (n)	HHcy mice (*n*)	p-value (t-test)
Dams	48.3 ± 2.0 [4]	250.9 ± 129.9 [4]	*0.021*
Sires	51.1 ± 11.8 [4]	326.6 ± 191.4 [4]	*0.023*
Neonate ♀, 6	57.72 ± 1.4 [3]	79.40 ± 5.5 [2]	*0.006*
Neonate ♂, 6	52.50 ± 3.0 [2]	60.85 ± 2.2 [3]	*ns*
Juveniles ♀, 30	45.61 ± 2.1 [2]	81.06 ± 9.5 [3]	*0.016*
Juveniles ♂, 30	51.29 ± 22.6 [2]	98.73 ± 18.8 [2]	*0.018*

### 3.2 Dietary-induced hyperhomocysteinemia causes breeding complications

The pregnancies of HHcy couples were characterized by significantly longer interpregnancy intervals. The total offspring from four pairs over three litters in each group were 69 and 72 for HHcy and control groups, respectively, showing no statistically significant difference. Although the litter sizes were similar, there was an almost 10-fold increase in intrauterine mortality in HHcy (mean number of stillbirths 1.5 vs. 14.2, *p* = 0.005, in control and HHcy pairs, respectively) (Supplementary Table S2). Stillborn fetuses’ development was arrested in the late second or third week of pregnancy (Supplementary Figure S3). HHcy pups exhibited a lower survival percentage (mean 80.8 ± 15.0 vs. 51.9 ± 22.2 [%], *p* = 0.015) in the following postnatal days.

### 3.3 Pups of the HHcy parents have elevated urine tHcy levels and reduced body weight

Mean urinary tHcy [µM] in HHcy female (but not male) pups was significantly elevated on PND 6. Following weaning, the high-Met diet led to significantly increased urinary tHcy levels in young mice of both sexes at 30 days (1.26- and 1.85-fold at PND 6 and PND 30; *p* = 0.028 and *p* < 0.0001, respectively) (Table 1). Additionally, HHcy pups showed significantly reduced birth weight. Female offspring exhibited significantly lower weight throughout their lives, while in males, the reduction was not significant after sexual maturity was reached (PND 40) (Supplementary Table S2; Supplementary Figures S4, S5).

### 3.4 HHcy alters locomotor behavior and activity in the offspring

**FIGURE 2:**
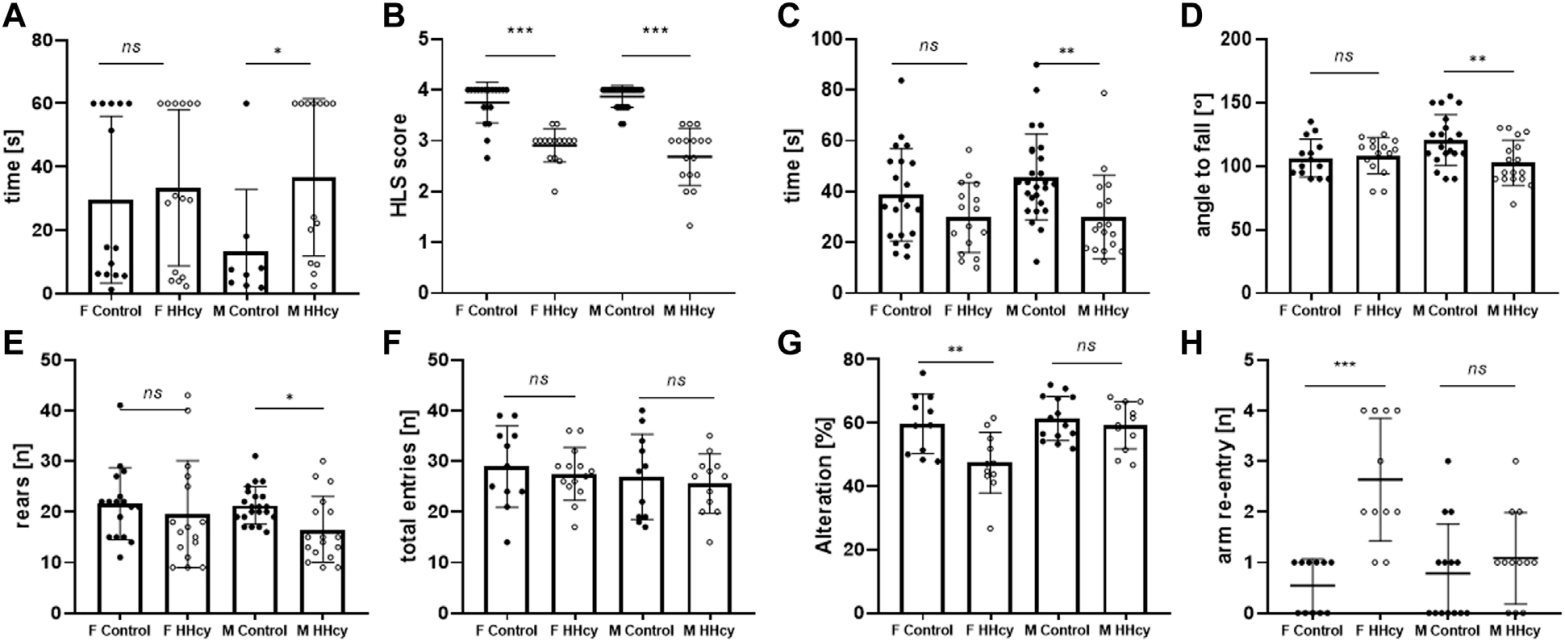
Sex-specific effects of the high-Met diet on the neuromotor and cognitive performance of neonatal mice. **(A)** Surface righting test [time of righting(s)], PND 5, ANOVA *p < 0.05. **(B)** Hindlimb suspension test (score), PND 6, ANOVA ***p < 0.0001. **(C)** Hindlimb suspension test [suspension time(s)], PND 6, ANOVA *p < 0.05. **(D)** Grip strength test [angle to fall (°)], PND 13, ANOVA **p < 0.01. **(E)** Cylinder test [number of rears (n)], PND 32, ANOVA *p < 0.05. **(F)** Y-maze [total entries (n)], PND 30, ANOVA ns *p* > 0.05. **(G)** Y-maze (% alteration), PND 30 ANOVA **p < 0.005. **(H)** Y-maze [arm re-entry (n)], PND 30 ANOVA ***p < 0.0001.

HHcy male pups displayed a significantly weaker surface righting reflex, taking longer to right compared to control pups (36.7 ± 24.9 *n* = 8 vs. 13.4 ± 19.5 *n* = 14, *p* = 0.0374). In females, there was no difference in the time spent to right between groups (29.6 ± 26.3 *n* = 14 vs. 33.4 ± 24.7 *n* = 15, *p* > 0.05) (Figure 2A). On the other hand, HHcy pups exhibited significantly lower HLS scores for both females (2.91 ± 0.32 *n* = 15 vs. 3.75 ± 0.40 *n* = 19, *p* < 0.0001) and males (2.69 ± 0.56 *n* = 17 vs. 3.87 ± 0.22 *n* = 26, *p* < 0.0001), indicating neuromuscular dysfunction due to the high-Met diet (Figure 2B). However, HLS time decreased significantly for HHcy males (30.0 ± 16.5 *n* = 17 vs. 45.8 ± 16.8 *n* = 26, *p* = 0.0061) but not for HHcy females (29.8 ± 13.8 *n* = 16 vs. 39.7 ± 18.2 *n* = 21, *p* > 0.05), indicating impaired muscle strength in HHcy males only (Figure 2C). No difference in ambulation transmission on PND 9 was observed between control and HHcy groups (*p* > 0.05).

On PND 13, mice underwent four-paw strength testing. HHcy males exhibited a lower angle of falling off the rotating mesh (102.9 ± 17.7 *n* = 17 vs. 120 ± 19.9 *n* = 20, *p* = 0.05), indicating a significant impairment in grip strength compared to controls. No change in performance was observed in females (108.4 ± 14.1 *n* = 15 vs. 106.3 ± 14.9 *n* = 14, *p* > 0.05) (Figure 2D).

HHcy males showed significantly decreased spontaneous activity in the beaker test, measured by the number of rears (16.5 ± 6.5 *n* = 17 vs. 21.3 ± 4.3 *n* = 21, *p* = 0.046), while female activity did not differ significantly (19.6 ± 10.6 *n* = 16 vs. 21.6 ± 7.12 *n* = 17, *p* > 0.05) (Figure 2E).

### 3.5 HHcy alters cognition behavior in female offspring only

Recording the spontaneous alternation behavior in a Y-maze assessed spatial working memory performance. The total arm entries did not significantly differ between HHcy and control groups (Figure 1F, *p* > 0.05). However, only HHcy females showed significantly decreased spontaneous alteration (47.4 ± 9.6 *n* = 11 vs. 59.7 ± 9.4 *n* = 11, *p* = 0.024) (Figure 2G) and a significantly higher number of re-entries compared to controls (2.64 vs. 0.55, *p* < 0.0001) (Figure 2H). These findings indicate that HHcy impairs short-term memory in females but not in males.

### 3.6 HHcy affects brain parameters in female offspring only

Brain weight (0.377 ± 0.015 *n* = 9 vs. 0.389 ± 0.019 *n* = 13, (g), *p* < 0.05) and the whole brain horizontal length (8.72 ± 0.48 *n* = 4 vs. 9.30 ± 0.19 *n* = 5, (mm), *p* < 0.05) were significantly reduced only in HHcy females compared to control females. Although the size of the hippocampus and the thickness of the granule cell layer (DGEC) were not affected by HHcy in either sex, the thickness of the pyramidal cell layer within the CA1 region was reduced in HHcy females (41.66 ± 1.91 *n* = 4 vs. 47.89 ± 2.40 *n* = 5, (µm), *p* < 0.005). The results of morphometric assessments are presented in Table 2. These findings indicate that HHcy affects brain morphology in females but not in males.

**TABLE 2:** Measurements of brain parameters in offspring from control and HHcy parents, end of study (PND40). Brain sections between bregma −1.80 mm and −2.30 mm were used to assess brain parameters. Significance between control and HHcy in each sex was calculated using Student’s t-test.

	Female control (*n*)	Female HHcy (*n*)	p-value	Male control (*n*)	Male HHcy (n)	*p*-value
Brain weight (g)	0.389 ± 0.019 [13]	0.377 ± 0.015 [9]	*0.04*	0.392 ± 0.011 [13]	0.385 ± 0.020 [15]	*ns*
Brain vertical length (mm)	5.80 ± 0.29 [5]	5.42 ± 0.41 [4]	*ns*	5.66 ± 0.23 [5]	5.64 ± 0.10 [4]	*ns*
Brain horizontal length (mm)	9.30 ± 0.19 [5]	8.72 ± 0.48 [4]	*0.039*	9.14 ± 0.43 [5]	9.14 ± 0.37 [4]	*ns*
Brain area (mm^2^)	42.38 ± 2.85 [5]	37.18 ± 4.46 [4]	*ns*	40.67 ± 3.56 [5]	40.45 ± 1.89 [4]	*ns*
Hippocampus vertical length (mm)	1.128 ± 0.073 [5]	1.118 ± 0.044 [4]	*ns*	1.145 ± 0.138 [5]	1.161 ± 0.026 [4]	*ns*
Hippocampus horizontal length (mm)	2.301 ± 0.078 [5]	2.317 ± 0.087 [4]	*ns*	2.286 ± 0.138 [5]	2.352 ± 0.092 [4]	*ns*
Hippocampus area (mm^2^)	2.04 ± 0.20 [5]	2.04 ± 0.13 [4]	*ns*	2.10 ± 0.20 [5]	2.16 ± 0.11 [4]	*ns*
DGEC stratum granulosum thickness (µm)	66.73 ± 4.85 [5]	65.08 ± 1.76 [4]	*ns*	72.52 ± 2.46 [5]	69.39 ± 3.09 [4]	*ns*
CA1 stratum pyramidale thickness (µm)	47.89 ± 2.40 [5]	41.66 ± 1.91 [4]	0.004	44.77 ± 3.23 [5]	43.23 ± 1.54 [4]	*ns*

### 3.7 High-Met diet upregulates mTOR signaling and inhibits autophagy in offspring cortexes

We found that the proteins involved in autophagy were dysregulated in HHcy mouse brains. Regulators of autophagosome assembly Becn1 and Atg5 were significantly downregulated in HHcy male brains, while in HHcy females, only Atg5 was downregulated, whereas Atg7 was upregulated. P62 accumulation was significantly elevated only in HHcy females. Because autophagy is mainly regulated by mTOR, we also quantified effects of HHcy on mTOR accumulation and its activation. In the cortex of HHcy females, mTOR exhibited a notable increase; however, its active form, phosphorylated at serine 2,448, remained unaffected. In HHcy males, we observed significant mTOR activation (Supplementary Figures S6A, B). The list of tested proteins is given in Supplementary Table S4.

## 4 Discussion

The current study aimed to explain the consequences of chronic parental HHcy on offspring, with a particular focus on elucidating the associations between observed sex-dependent cognitive and muscular deficits and the dysregulation of mTOR-autophagy, alongside alterations in the brain structure. In our study, we assessed cognition using the Y-maze test in mice at 1 month of age. Spontaneous alternation, an indicator of spatial working memory, was diminished exclusively in HHcy females, with no impairment observed in males. Intact working memory in mice typically leads to a preference for entering less recently visited arms. In our study, HHcy females also demonstrated an increased propensity to revisit the previously explored arm. These impairments may be explained by morphometric changes we found only in HHcy female brains. While the decreased brain weight and horizontal length in HHcy females may be attributable to their reduced body weight, the thinning of the stratum pyramidale in the CA1 region was observed exclusively in HHcy females, despite no discernible differences in hippocampal size compared to controls. The hippocampus, particularly in the CA1 region, is vital for numerous cognitive functions such as memory consolidation, spatial memory, and learning (Eichenbaum, 2004). Disrupted neuronal ultrastructure in this region was also observed in rats’ offspring from HHcy dames (Shcherbitskaia et al., 2021). In addition to the observed structural changes in HHcy females’ brains, we also noted disturbances in autophagy. Congenital autophagy disorders are associated with neurometabolism and childhood-onset neurological diseases (Teinert et al., 2020). The expression of autophagy genes is essential for the proper development of the CNS (Wu et al., 2013) and neurogenesis during embryo development (Kuma et al., 2017). Defective autophagy leads to p62 accumulation, which was observed in female HHcy mice in this study, which might also have contributed to the poorer performance in the Y-maze test. In contrast to females, HHcy males did not exhibit cognitive impairments. We also noticed that HHcy did not affect the brain structure in male offspring. In our study, male neonates exposed to HHcy demonstrated diminished strength, indicated by prolonged righting time on PND 5, decreased HLS time on PND 6, a smaller falling angle in the GS test on PND 13, and fewer rears in the beaker test at 1 month of age. Previous research links HHcy to impaired physical performance and muscle force generation in adult mice (Veeranki and Tyagi, 2013; Veeranki et al., 2015). Atg7 is required in skeletal muscle for development, basal homeostasis, and adaptation. Loss of Atg7 in embryonic or adult skeletal muscle caused loss of muscle mass and strength (Masiero et al., 2009). We found that Atg7 was differently regulated in both sexes by HHcy. While sporadic activation of mTORC1 is essential to trigger muscle hypertrophy, prolonged activation of mTOR is detrimental, resulting in muscle atrophy and impaired function (Goodman, 2019). In HHcy males, there was a significant elevation in phospho-mTOR, accompanied by the downregulation of Atg7. This, in conjunction, may elucidate the observed muscle strength impairments exclusively in this group. Loss of Atg5 in the cortex that was found in our studies may cause impaired morphology of cortical neurons (Lv et al., 2014). In this context, the downregulation of Atg5 in HHcy juveniles’ cortexes may correlate with neuromuscular impairments we found in neonates of both sexes in the HLS test. We hypothesize that the observed behavioral impairments are the result of altered embryogenesis due to a dysfunctional placenta. Because HHcy is a risk factor for vascular diseases examined by our group and other investigators in humans, mouse models, and cell culture (Devlin et al., 2005; Perła-Kaján et al., 2008; Kim et al., 2011; Huang et al., 2012; Gurda et al., 2015), we hypothesize that elevated Hcy may be a potential marker of placental vascular disease. It was reported before that unusually high Hcy levels are linked in humans to pregnancy-related hypertension disorders and unfavorable pregnancy outcomes, including spontaneous abortions (Chamotra et al., 2020), fetal growth restriction, and preeclampsia (Chen et al., 2018; Gaiday et al., 2018). The mechanism by which HHcy may affect embryo development is insufficient remodeling of the spinal arteries and impaired angiogenesis in the placenta (Burton and Jauniaux, 2018). Underdeveloped or damaged placenta may explain the phenomenon of intrauterine mortality and lower survival rate in neonates in the HHcy group found in this study, as well as abnormal brain development. Recent studies on rodent models have shown placenta and fetus weight decrease in the HHcy rats on embryonic day 20 (Arutjunyan et al., 2020). HHcy and oxidative stress impair the endothelial cell layer, which leads to endothelial nitric oxide synthase downregulation and a significant reduction in endothelium‐dependent vasorelaxation (Cheng et al., 2009). Autophagy and apoptosis are two crucial, interconnected processes in the placenta and developing embryo that are often influenced by oxidative stress. Khayati et al. (2017), Witucki et al. (2023), and Yan et al. (2021) showed that HHcy inhibits autophagy. Those aspects combined might elucidate our observation that dietary HHcy not only causes a significant increase in intrauterine mortality but also contributes to body weight reduction and neuromotor and cognitive deficits in offspring born to HHcy parents.

Summarizing, we demonstrated that a high-Met diet impairs memory and cognition in female juveniles and weakens muscle strength in male pups. This effect may be caused by abnormal placenta development and function, affecting early neurogenesis, the dysregulation of autophagy-related pathways in the mouse cortex, or epigenetic mechanisms of gene regulation in embryonic development triggered by HHcy (Vujkovic et al., 2009).

## Data Availability

The raw data supporting the conclusion of this article will be made available by the authors, without undue reservation.
