# Supplementary material for: Diet-induced hyperhomocysteinemia causes sex-dependent deficiencies in offspring musculature and brain function
Source: Front Cell Dev Biol. 2024 Mar 15;12:1322844. doi: 10.3389/fcell.2024.1322844 (PMC10979824; doi:10.3389/fcell.2024.1322844)
Supplement: Supplementary file 1 [file Presentation1.pdf]

Supplementary Table ST1. Hindlimb suspension score guidance for behavioral performance.

score	scoring criteria
4	Normal hindlimb separation with tail raised
3	Weakness is apparent and hindlimbs are closer together but they seldom touch each other
2	Hindlimbs are close to each other and often touching
1	Weakness is apparent and the hindlimbs are almost always in a clasped position
0	Constant clasping of the hindlimbs with the tail lowered or failure to hold onto the tube for any period of time

Supplementary Table ST2. Breeding outcomes in control and HHcy mice (4 pairs in each group) across three consecutive litters.

	1st litter		2nd litter		3rd litter		total of 3 litters		
	Control	HHcy	Control	HHcy	Control	HHcy	Control	HHcy	P value
Mean interpregnancy interval (days)	1.3	4.3	3.7	6.3	3.7	5.8	2.9±1.8	5.4±1.8	*0.0044***
total born, n	22	28	23	22	24	22	69	72	*ns*
dead fetuses, n	1	3	0	1	0	6	1	10	*0.0054**
born dead, %	4.6	10.7	0	4.6	0	27.3	1.5	13.9	*0.0054**
Alive PND>2, n	14	8	21	16	21	12	56	36	*0.015***
Survival, %	63.6	28.6	91.3	72.7	87.5	54.5	80.8±15.0	51.9±22.2	*0.015***
* Fisher test, ** T-test, HHcy vs. control									

Supplementary Table ST3. Mean body weight (g) of offspring born to control and HHcy mothers throughout the experiment.

**offspring sex and age (days)**	Offspring (n) from control mothers	Offspring (n) from HHcy mothers	P value (T-test)
Neonate ♀and ♂, 1	1.31±0.10 (21)	1.23±0.09 (39)	*0.0038*
Neonate ♀and ♂, 6	2.63±0.19 (32)	2.39±0.46 (16)	*0.0250*
Juveniles ♀, 30	13.29±1.29 (11)	11.57±2.33 (11)	*0.0440*
Juveniles ♂, 30	14.23± 2.22(14)	10.07± 1.47(13)	*0.00002*
Young adult ♀, 40	16,57±0.66 (13)	16,01±0.69 (13)	*0.045*
Young adult ♂, 40	19.45±1.29 (13)	18.72±2.23 (15)	*ns*

Supplementary Table ST4. List of proteins tested by Western Blot (WB) in mouse cortex, including name, UniProtKB entry numbers, and calculated (UniProt data) and observed (Western Blot analysis) protein mass.

**Recommended protein name**	Short Protein name	UniProtKB entry	calculated mass (KDa)	Observed mass (KDa)
Beclin-1	BECN1	O88597	51.589	62
Autophagy protein 5	ATG5	Q99J83	32.402	55
Ubiquitin-like modifier-activating enzyme ATG7	ATG7	Q9D906	77.520	75
Sequestosome-1	P62	Q64337	48.163	62
Serine/threonine-protein kinase mTOR	MTOR	Q9JLN9	288.789	290
Glyceraldehyde-3-phosphate dehydrogenase	GAPDH	Q5XJ10	35.784	37

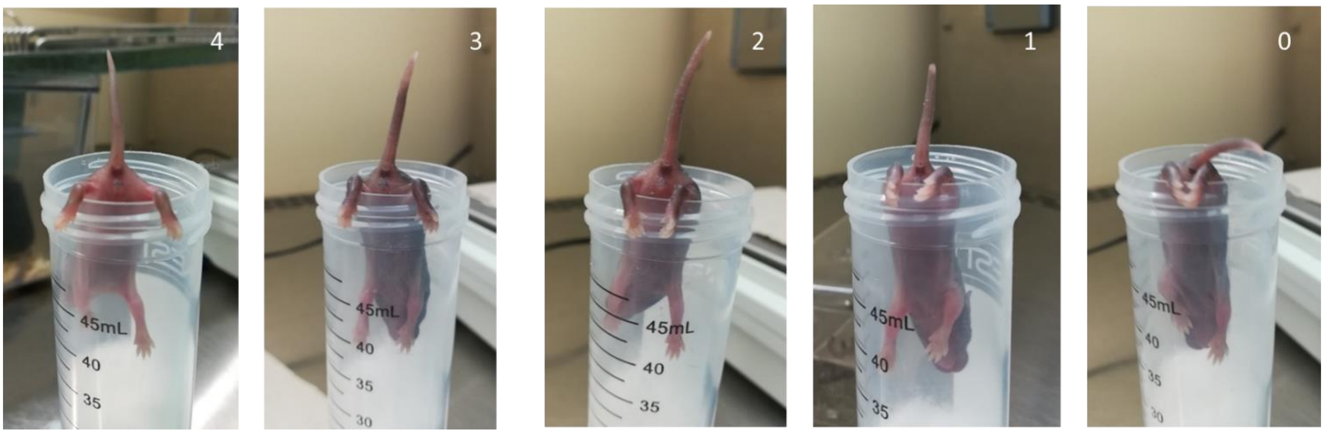

Supplementary Figure SF1. Representative pictures of mice demonstrating the possible posture in Hindlimb suspension test. Example individual score is noted above the pictures.

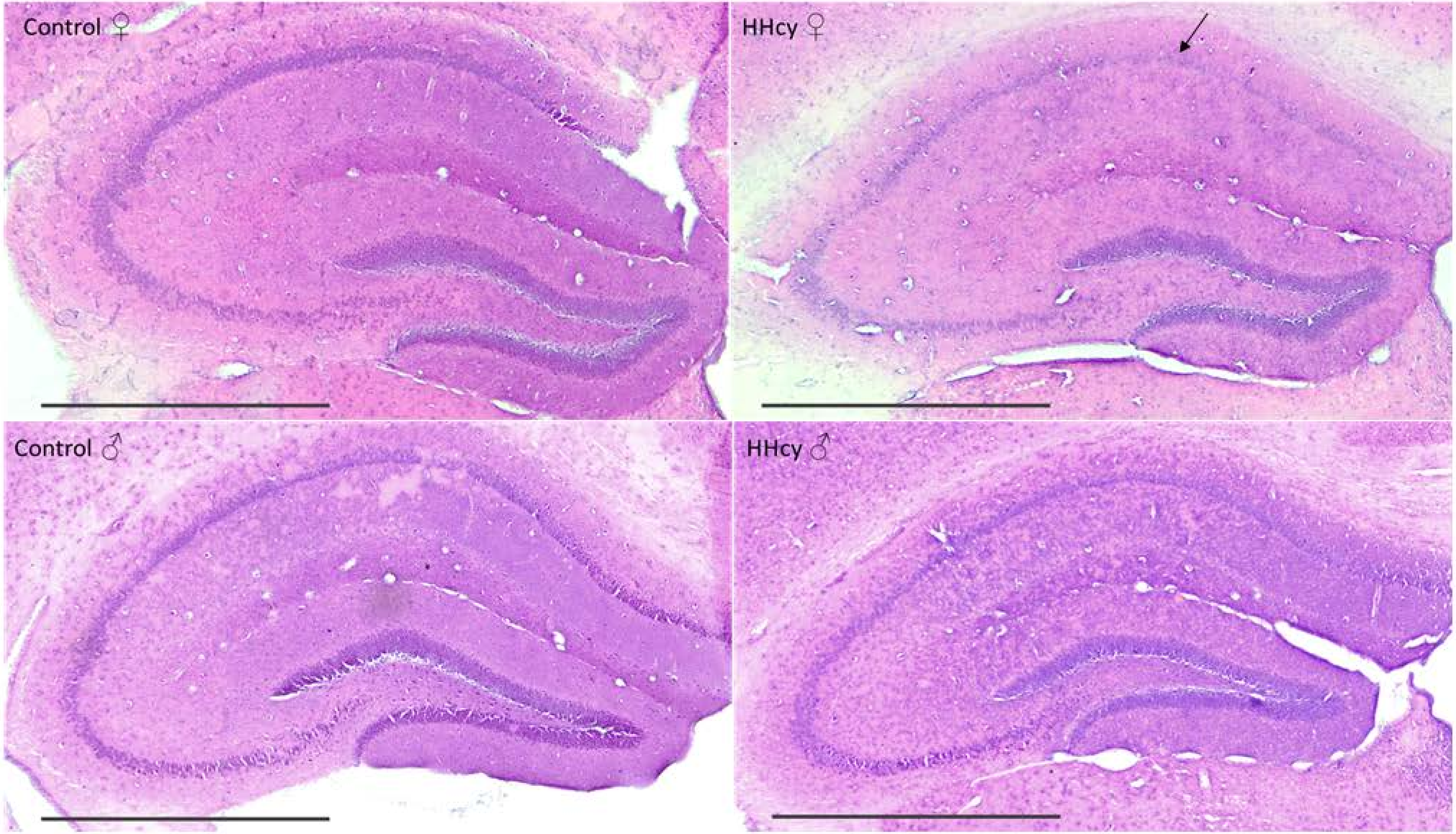

Supplementary Figure SF2. Representative micrographs of hematoxilin-eosin stained coronal hippocampal sections from control and HHcy offspring. Arrow indicates the reduced thickness of the layer of pyramidal cells in the CA1 region of the hippocampus in HHcy females. Scale bar 1 mm.

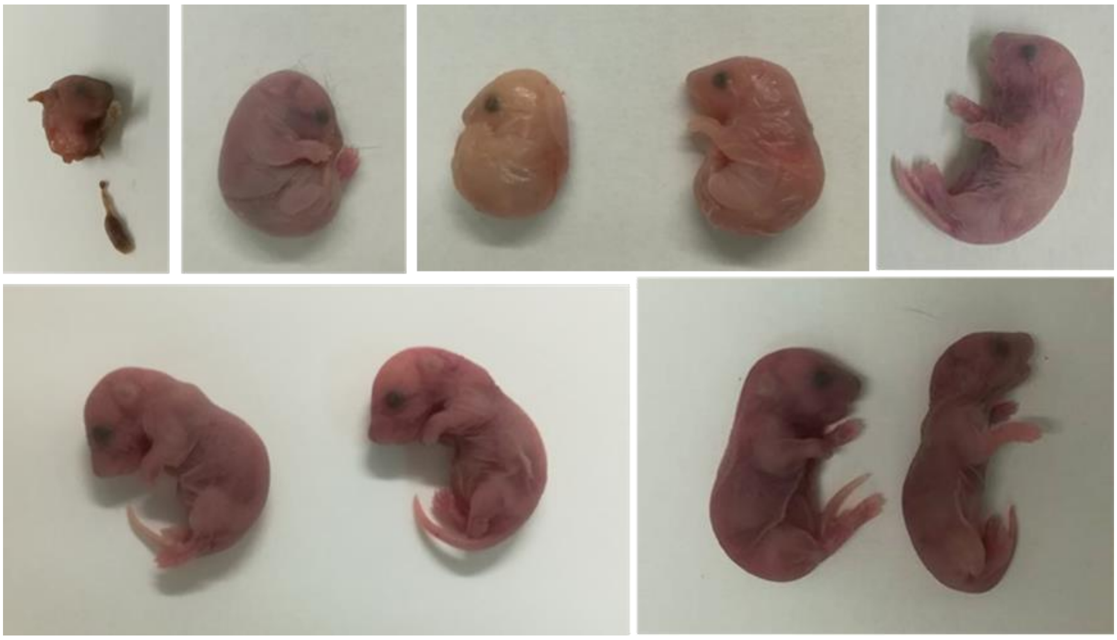

Supplementary Figure SF3. Example pictures of the stillborn fetuses in the HHcy group. Fetuses development arrested in late 2nd and 3rd week of pregnancy.

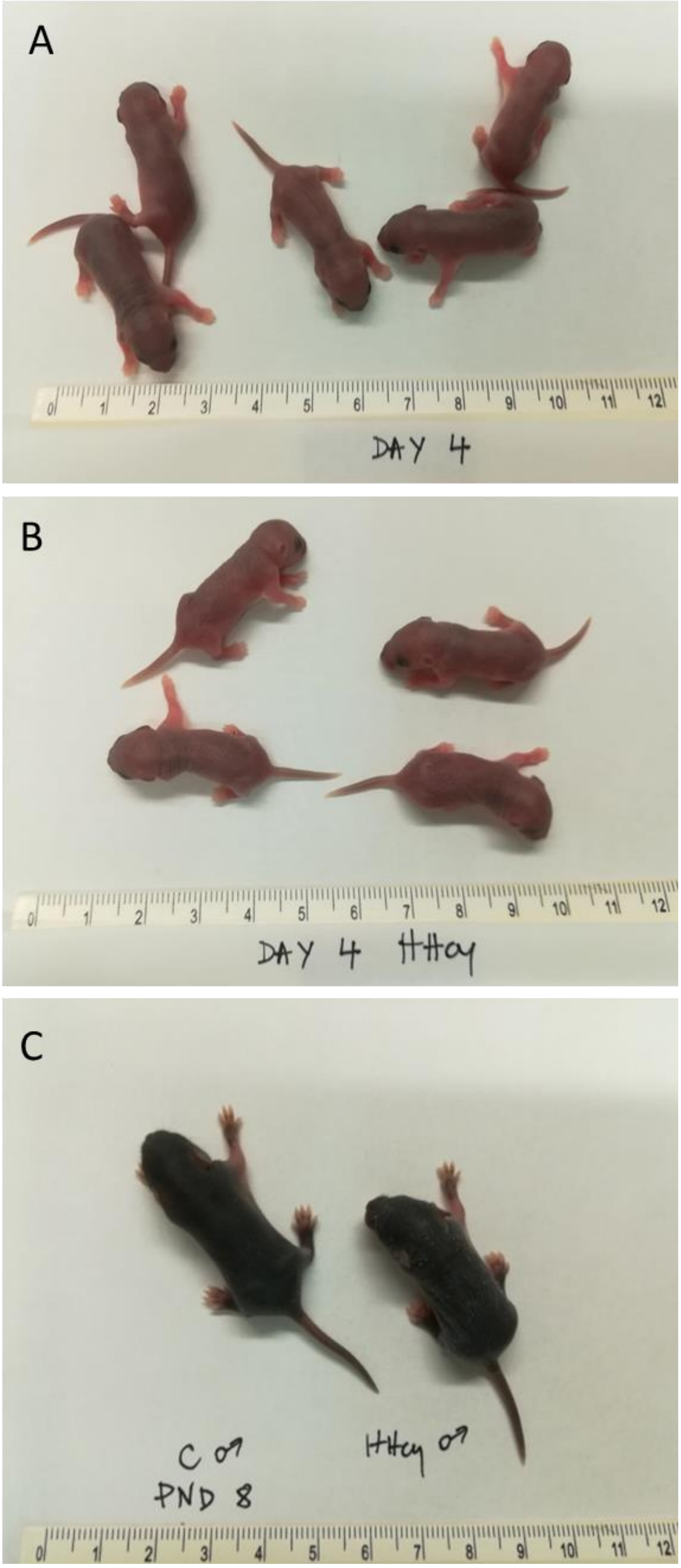

Supplementary Figure SF4. Example pictures comparing control and HHcy neonates' sizes. A-control mice PND4, B-HHcy mice PND4, C-control and HHcy mouse PND8.

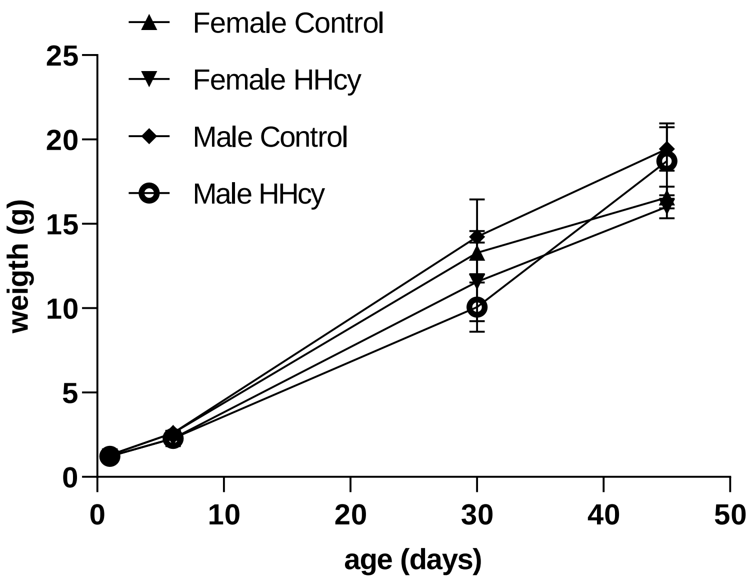

Supplementary Figure SF5. The effect of HHcy on offspring weight reduction throughout the experiment. The numerical data are presented in ST3.

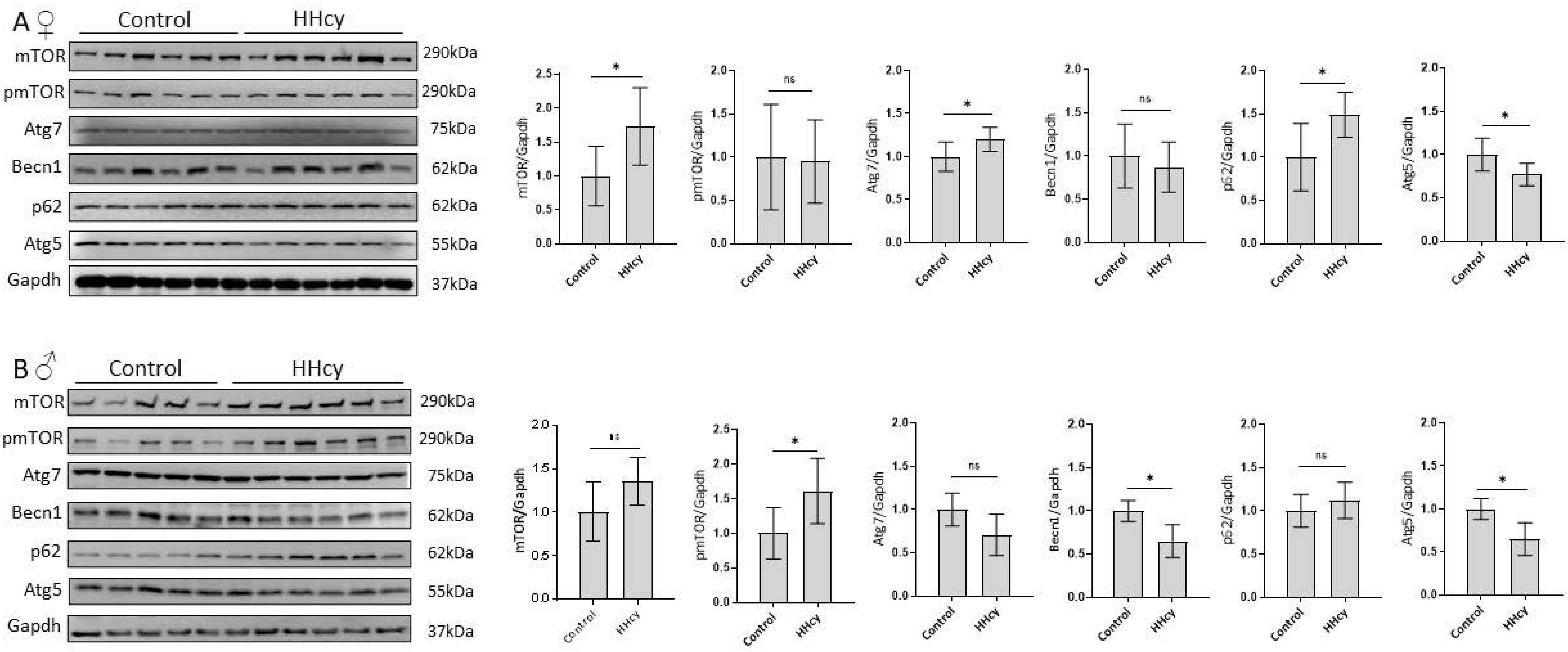

Supplementary Figure SF6. Sex-specific effects of a high-Met diet on mTOR and autophagy-related proteins in the cortex of 1-month-old offsprings. Representative Western blot images, used for protein quantification, and corresponding bar graphs are presented.
Panel A. Specific dysregulation of proteins in females, T-test ***P < 0.05***;
Panel B. Specific dysregulation of proteins in males, T-test ***P < 0.05***
